# Strong Coupling Møller–Plesset Perturbation Theory

**DOI:** 10.1021/acs.jctc.5c00055

**Published:** 2025-03-31

**Authors:** Yassir El Moutaoukal, Rosario R. Riso, Matteo Castagnola, Enrico Ronca, Henrik Koch

**Affiliations:** † Department of Chemistry, Norwegian University of Science and Technology, 7491 Trondheim, Norway; ‡ Department of Chemistry, Biology and Biotechnology, University of Perugia, Via Elce di Sotto, 8, 06123 Perugia, Italy

## Abstract

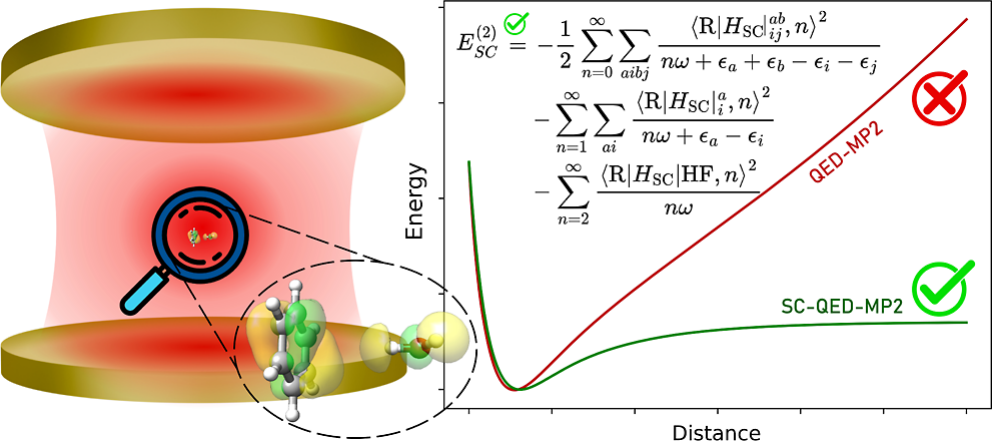

Perturbative approaches are methods to efficiently tackle many-body problems, offering both intuitive insights and analysis of correlation effects. However, their application to systems where light and matter are strongly coupled is nontrivial. Specifically, the definition of suitable orbitals for the zeroth-order Hamiltonian represents a significant theoretical challenge. While reviewing previously investigated orbital choices, this work presents an alternative polaritonic orbital basis suitable for the strong coupling regime. We develop a quantum electrodynamical (QED) Møller–Plesset perturbation theory using orbitals obtained from the strong coupling QED Hartree–Fock. We assess the strengths and limitations of the different approaches with emphasis on frequency and coupling strength dispersions, intermolecular interactions and polarization orientational effects. The results show the essential role of using a consistent molecular orbital framework in order to achieve an accurate description of cavity-induced electron−photon correlation effects.

## 1 Introduction

Strong coupling between electromagnetic vacuum fluctuations and matter allows for noninvasive engineering of molecular properties.^1−6^ To achieve such a regime, experimentalists couple molecules with optical devices able to confine the electromagnetic fields in small quantization volumes.^7−9^ When molecular excitations interact with the quantized fields, hybrid states called molecular polaritons are formed.^10−12^ Such states display unique features that can be adjusted by tuning the properties of the quantized field.^13^ Potential applications of polaritonic chemistry range from the modification of molecular absorption and emission spectra to the potential catalysis of chemical reactions.^14−20^ While experimental efforts keep advancing into the ultrastrong coupling regime, a theoretical comprehension of the experimental results is still necessary.^21−26^ Modeling the light-matter interplay requires the use of QED theory in order to capture the correlation effects between electrons and photons.^27−32^ Several quantum chemistry ab initio methodologies have been extended to QED environments, such as the quantum electrodynamical density functional theory (QEDFT)^33−36^ and the quantum electrodynamical coupled cluster (QED-CC).^37−41^ Despite its computational affordability, the accuracy of QEDFT relies on an electron-photon correlation functional that is challenging to model,^36^ while the more accurate QED-CC exhibits a steep computational scaling with system size.^42^ Perturbative methodologies are reliable alternatives to estimate correlation at a cheaper computational cost while providing, at the same time, an intuitive understanding of the complex interplay between the components of the many-body system. Inside an optical cavity, perturbative approaches can either be obtained by excluding the field-dependent terms from the unperturbed Hamiltonian, in line with what is reported by Haugland et al.,^43^ or by retaining the mean-field effects of the cavity in the zeroth-order Hamiltonian. Bauer et al.^44^ reported an implementation of the first QED versions of the second order Møller–Plesset methodology (MP2) and the algebraic diagrammatic construction for the polarization propagator (ADC(2)). Specifically, the method are built starting from two possible reference states: the standard nonpolaritonic Hartree–Fock state (QED(np-HF)-MP2 and QED(np-HF)-ADC(2)), and the QED Hartree–Fock (QED-HF) wave function (QED-MP2 and QED-ADC(2)). These approaches seem to accurately describe light-matter states while incorporating a significant part of many-body correlation. These are surprising findings as Haugland et al.^28^ demonstrated that the QED-HF molecular orbitals display unphysical properties, such as their lack of origin invariance for charged molecular systems due to an incorrect construction of the QED-Fock operator posing issues when developing post-HF perturbation theories.

The polaritonic molecular orbital problem for QED environments was addressed by Riso et al.^45^ introducing a novel ab initio approach named strong coupling quantum electrodynamics Hartree–Fock (SC-QED-HF) theory. This model not only provides fully consistent molecular orbitals by dressing the electrons with the cavity photons, but is also able to capture to some extent electron-photon correlation already at the mean-field level. Recent improvements in the convergence of SC-QED-HF by means of second order algorithms^46^ prompted us to develop of a Møller–Plesset perturbation theory starting from this alternative reference wave function. We denote this method as SC-QED Møller–Plesset perturbation theory. Another perturbative approach based on a wave function parametrization similar to the SC-QED-HF was recently published, namely the Lang-Firsov Møller–Plesset scheme (LF-MP2).^47^ The main difference between the two methods is that the diagonal Lang-Firsov transformation in LF-MP2 is not performed in a basis that diagonalizes the dipole operator. Our findings demonstrate that the developed SC-QED-MP2 accurately reproduces the field-induced electron-photon correlation effects by capturing them already at the mean-field level. This is especially the case as the light-matter coupling increases due to the exactness of the reference wave function in the infinite coupling limit. Moreover, because of the non size-intensivity of the zeroth-order Hamiltonian in QED-MP2 theory, unphysical behavior in the long-range regime between two molecular systems is observed. The same issue emerges for the LF-MP2 method, suggesting that the choice of basis in QED ab initio approaches is a delicate matter.

This paper is organized as follows. In Section 2, we describe the different choices for the zeroth-order Hamiltonian, deriving the energy expressions for QED-MP2, QED(np-HF)-MP2, and SC-QED-MP2 theory. In this Section we also show the differences between the SC-QED-MP2 and LF-MP2 approaches. In Section 3, we compare the performance of methodologies, focusing on coupling and frequency dispersions, intermolecular interactions, and cavity polarization orientational effects. Finally, our concluding remarks are presented in Section 4.

## 2 Theory

The interaction between light and matter inside an optical cavity can be modeled using the single-mode Pauli-Fiertz Hamiltonian in the dipole approximation and length gauge^48−52^
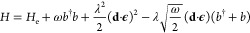
1where *b* and *b*^†^ annihilate and create photons of frequency ω and with polarization . The λ parameter represents the light-matter coupling strength for a field confinement volume *V* and relative permittivity for the medium within the cavity ϵ_r_
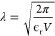
2while **d** is the molecular dipole operator^28^
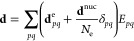
3with **d**^e^ being the electronic dipole operator and **d**^nuc^ the nuclear dipole moment of a system of *N*_e_ electrons. The second quantization formalism for the electrons has been adopted in eq 1 such that
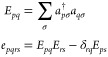
4with *a*_*p*σ_^†^ and *a*_*p*σ_ respectively create and annihilate an electron in the orbital *p* with spin σ. Finally, the electronic Hamiltonian in the Born–Oppenheimer approximation *H*_e_ is defined as^42^
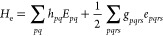
5where *h*_*pq*_ and *g*_*pqrs*_ are the one and two electron integrals. In the following, we label occupied orbitals in the HF reference with the letters *i*, *j*, *k*... while the virtual orbitals are labeled *a*, *b*, *c*... General orbital indices are labeled with *p*, *q*, *r*, *s*. In addition to the standard electronic terms, the strong coupling Hamiltonian in eq 1 has three additional field-induced contributions, i.e. the purely photonic Hamiltonian ω*b*^†^*b*, the bilinear light-matter term, , explicitly correlating the field and the electrons, and finally the dipole self-energy (DSE) term, , needed to ensure that the Hamiltonian is bound from below.^53^

Rayleigh–Schrödinger (RS) perturbative schemes rely on a partition of the full Hamiltonian into a zeroth-order unperturbed Hamiltonian, *H*^(0)^, whose eigenfunctions are known, and a perturbation.^54^ The perturbation *V* can be as complicated as needed to describe the physics of the overall system. When *H*^(0)^ is chosen to be the Fock operator from a mean-field theory, aimed at capturing the main physical properties of the system, we obtain the Møller–Plesset (MP) perturbation hierarchy.^55^ Accordingly, the perturbation *V* is defined such that when added to the “solvable” zeroth-order Hamiltonian *H*^(0)^, the full Hamiltonian is recovered
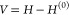
6and incorporates all the correlation effects of the many-body system. The definition of an appropriate zeroth-order Hamiltonian is critical to ensure that perturbative methodologies provide a reliable description of the system, particularly if only a few orders in perturbation theory are considered.^42^

In this Section, we first review different choices for QED Møller–Plesset perturbation theory.^44^ Then, we present the SC-QED-MP2 approach with the unperturbed Hamiltonian derived from the polaritonic mean field treatment of SC-QED-HF theory. Lastly, we review the LF-MP2 method with emphasis on the differences between this approach and the developed SC-QED-MP2 method.

### 2.1 QED-MP2

In the quantum electrodynamics Hartree–Fock method, the wave function is written as
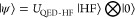
7which is composed of the bosonic vacuum |0⟩ and an electronic Hartree–Fock–Slater determinant, |HF⟩, where the low lying orbitals are occupied. The coherent-state transformation
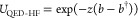
8depends on the factor  which is updated throughout the SCF procedure together with the orbitals and
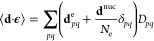
9where *D*_*pq*_ = ⟨HF|*E*_*pq*_|HF⟩ are the one-body density matrix elements. It is convenient to change the quantum picture by applying the transformation to the light-matter Hamiltonian in eq 1
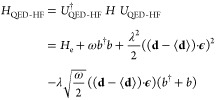
10such that the origin-independence becomes explicit. In this representation, the QED-HF wave function reads
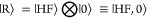
11Bauer et al.^44^ proposed the QED-HF Fock operator plus the field energy ω*b*^†^*b* as the zeroth-order Hamiltonian for the QED-MP2 approach. Additionally, we highlight that the expectation value of the dipole squared (≡⟨**d**·**ϵ**⟩^2^) should be included as well in the unperturbed Hamiltonian if not included in the perturbation *V*.^44^ The unperturbed Hamiltonian then reads
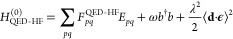
12where the Fock matrix elements are
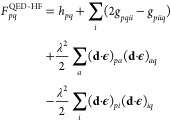
13We point out that the coherent-state transformation does not change the electronic Hamiltonian. This will not be the case for the SC-QED-MP2 theory, and a correct transformation of the Fock matrix elements will be important to ensure orbital origin invariance. For charged molecules, upon a shift **a** of the molecular system, the dipole integrals shift according to
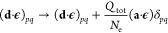
14where *Q*_tot_ is the total system charge. As a consequence, the Fock matrix elements in eq 13 are changed to
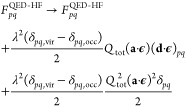
15and this effect prevents the orbitals and their energies to be origin invariant. Since the unperturbed Hamiltonian changes upon displacement of a charged molecular system, we expect an unphysical behavior of QED-MP2. Nonetheless, only rarely we do work with charged molecules and the problem can eventually be solved using the SC-QED-HF orbitals. However, a more severe problem of the Fock operator in eq 13 is the non size-intensivity. That is, for two systems A and B infinitely separated, the Fock operator is not equal to the sum of the two subsystems Fock operators
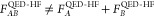
16

The orbital energies of the system *A*, instead, change if another system *B* is added in the cavity regardless of the distance between *A* and *B*. Upon insertion of molecule *B*, indeed, *F*_*A*_^QED-HF^ changes because the contribution from the nuclei of *B* needs to be added in the dipole operator. This might create problems when dealing with multicomponent systems. Nonetheless, we point out that the QED-HF method is size-extensive, i.e. the energy of a bipartite system where the subsystems A and B are far apart equals the sum of the individual subsystem energies. For this reason, QED-HF is unable to account for the cavity-induced non size-extensive effects.^56^

The zeroth to second QED-MP energy corrections are given by the expressions
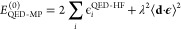
17
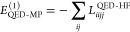
18
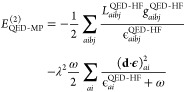
19

In the last equations, the redefined two electron integrals *g*_*pqrs*_^QED-HF^ and the integrals *L*_*pqrs*_^QED-HF^ are defined as
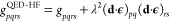
20
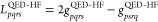
21while ϵ_*ai*_^QED-HF^, ϵ_*aibj*_^QED-HF^ showing in the denominators of the second order correction are
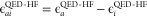
22
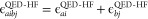
23where ϵ_*p*_^QED-HF^ are the orbital energies obtained from the diagonalization of the QED-HF Fock matrix in eq 13. Similarly to what happens with the standard MP2 scheme, the QED-HF energy is the sum of the zeroth and first-order energies. We point out that neglecting the contribution ∝⟨**d**·**ϵ**⟩^2^ in the zeroth-order Hamiltonian in eq 12 would have led to the wrong QED-HF energy. Thus, the first nonvanishing correction to the QED-HF energy occurs in second-order of perturbation theory. The QED-MP2 correction in eq 19 consists of two terms. The first term contains a contribution similar to the double excitations in the purely electronic MP2. However, it is important to highlight that the dipole contributions in *g*_*pqrs*_^QED-HF^ make the first term non size-extensive. The second term in eq 19, instead, stems from the bilinear term of the Hamiltonian. It consists of contributions from single excitations in the electronic Hilbert space , coupled with single excitations in the photonic Hilbert space  (double excitations in the polaritonic Hilbert space ). This term is size-extensive and partially cancels the DSE contribution to the QED-HF energy.^57^ Regarding size-intensivity, when changing the distance between two molecules that are already significantly far apart from each other, only the diagonal elements of the dipole matrix change significantly. Those elements enter the QED-MP2 energy correction in eq 19 through the orbital energies in the denominator as
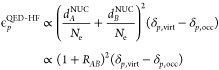
24where *R*_AB_ is the distance between the two molecules. Therefore, easy to see that the QED-MP2 energy correction vanishes as 1/*R*_AB_^2^.

### 2.2 QED-(Nonpolaritonic HF)-MP2

Even in the strong coupling regime, electron–electron correlation is dominating over the electron-photon one. It is therefore reasonable to substitute the QED-HF Fock matrix in eq 12 with the cavity free Hartree–Fock counterpart. Proceeding with this choice, we end up with the QED nonpolaritonic HF Møller–Plesset perturbative scheme,^44^ QED(np-HF)-MP2, with the zeroth-order Hamiltonian
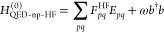
25and the Fock matrix elements are
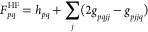
26

The reference wave function is again eq 11 and the zeroth-order Hamiltonian in eq 25 does not account for any cavity effect on the electronic system. For this reason, as the coupling between light and matter increases, we expect the accuracy of QED(np-HF)-MP2 to diminish because the field effects become progressively more significant, and their inclusion in the zeroth-order (mean-field) Hamiltonian becomes important. In this framework, the unperturbed Hamiltonian is both origin invariant and size-extensive, i.e. *H*_*AB*_^(0)^ = *H*_*A*_^(0)^+*H*_*B*_^(0)^ for largely separated *A* and *B*. Straightforwardly, when the two subsystems are infinitely far apart, the total energy reaches a plateau.

The zeroth to second order QED(np-HF)-MP energy corrections are given by the expressions
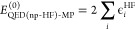
27
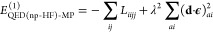
28
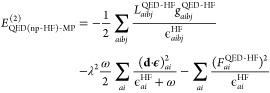
29where the Fock matrix elements *F*_*pq*_^QED-HF^, the two electron integrals *g*_*pqrs*_^QED-HF^ and *L*_*pqrs*_^QED-HF^ are defined respectively in eqs 13, 20 and 21. The definitions of *L*_*pqrs*_, ϵ_*ai*_^HF^ and ϵ_*aibj*_^HF^ are analogous to the ones in eqs 21–23. The correction up to the first order (with eq 28) of the zeroth-order energy gives the energy of the HF state in the cavity. This energy is higher compared to its polaritonic counterpart since the orbitals are not optimized including the DSE contribution. By comparing the second order energy correction in eq 29 with the expression from QED-MP2 theory, we notice that the two first terms change in the denominators with the cavity-free HF orbital energies. Moreover, an additional single electronic excitation contribution appears because the Brillouin theorem is not satisfied
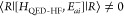
30

### 2.3 Strong Coupling QED-MP2

In the strong coupling quantum electrodynamics Hartree–Fock method, the wave function parametrization for the light-matter system is
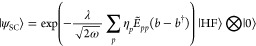
31where the tilde ∼ denotes integrals and operators in the basis that diagonalizes (**d**·**ϵ**)
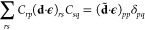
32where **C** is an orthonormal rotation matrix connecting molecular and dipole orbitals. The {η_*p*_} orbital specific coherent-state parameters in eq 31 account for the field rearrangement to orbital excitations and are variationally optimized for the ground state through the SCF procedure. Once again, it is convenient to change the quantum picture before partitioning the Hamiltonian into *H*^(0)^ and *V*. We apply the SC-transformation
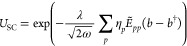
33to the Pauli-Fierz Hamiltonian in eq 1
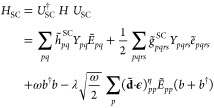
34where we defined the η-shifted dipole integrals as
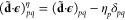
35The reference wave function |R⟩ is again defined in eq 11. The redefined SC one and two electron integrals entering in eq 34 read as
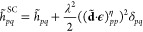
36
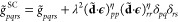
37while, the *Y*_*pq*_ and *Y*_*pqrs*_ photonic operators are defined as follows
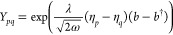
38
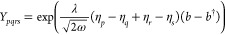
39

In line with the previous approaches, we define the zeroth-order Hamiltonian as
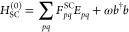
40

For this approach, there is no need to add the expectation value of the dipole squared contribution because the SC-transformation in eq 33 shifts the bosonic operators linearly with the {η_*p*_} parameters. These contributions are already reabsorbed in the SC-redefined integrals in eqs 36 and 37. This is not the case for the previous methods where the shift brought by the QED-HF transformation in eq 8 is ∝⟨**d**·**ϵ**⟩. Furthermore, as mentioned earlier, the effect of the SC-transformation in eq 33 on the Fock matrix elements
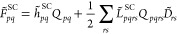
41is nontrivial due to the introduction of the Gaussian factors
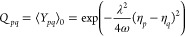
42
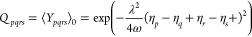
43that carry the ω-correlation captured at the mean field level. The integrals  read
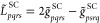
44while the Fock matrix elements in eqs 40 and 41 are connected by
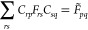
45where the canonical to dipole basis transformation **C** is defined in eq 32. The SC-QED-HF Fock is fully origin invariant as any displacement of the system (i.e., a change of the diagonal elements of ) is readily reabsorbed by an appropriate change of the optimal {η_*p*_} parameters. Moreover, the Fock matrix elements in eq 41 are also size-intensive as demonstrated in ref (45). The orbital specific coherent-state transformation in eq 31 inherently introduces correlation between electrons and photons, with significant implications on the perturbative energy corrections. The zeroth and first order energy corrections read
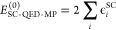
46
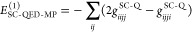
47and, once again, their sum leads to the SC-QED-HF energy. With ϵ_*p*_^SC^ we refer to the orbital energies obtained from the diagonalization of the SC Fock matrix in eq 41. The two electron integrals in the first order correction obtained from the dipole to canonical basis transformation of the integrals in eq 37 using the same **C** matrix used to change basis of the Fock matrix
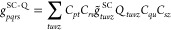
48with inclusion of the Gaussian factors *Q*_*pqrs*_.

The SC-QED Møller–Plesset second order energy correction is given by
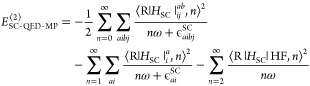
49where the index *n* refers to the number of photons in the respective excited determinant and
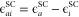
50
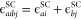
51

For a more detailed and explicit derivation of eq 49 we refer the reader to the Supporting Information where we also derive the more general perturbation theory for multiple sets of bosons and modes coupled to an electronic Hamiltonian. We highlight that the Hamiltonian now connects |HF, 0⟩ and determinants that include more than one photon. The excitations contributing to the second-order energy correction can be divided into three classes:

1. double excitations in the electronic reference with an arbitrary photon number |_*ij*_^*ab*^,*n* ≥ 0⟩. The contribution for *n* = 0 is equivalent to the first term in eq 19 while all the other terms are SC-QED-MP2 specific;
2. single excitations in the electronic reference with a photon number larger than zero |_*i*_^*a*^,*n* ≥ 1⟩. The contribution for *n* = 0 is zero because of the SC-QED-HF Brillouin condition in the orbital part
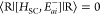
52The |_*i*_^*a*^,1⟩ term incorporates the light-matter bilinear contribution;
3. excitations in the field part only |HF, *n* ≥ 2⟩. These terms are specific to SC-QED-MP2. Their presence demonstrates that in the SC-QED-MP2 scheme photons and electrons are treated on an equal footing. The contribution for *n* = 1 is null because of the SC-QED-HF Brillouin condition in the photonic part
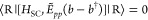
53

### 2.4 Lang–Firsov MP2

Recently, a Møller–Plesset perturbative approach up to the fourth order and based on a wave function similar to the SC-QED-HF one has been published.^47^ The LF-HF wave function parametrization reads
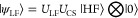
54where the transformations are given by
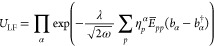
55
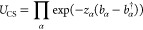
56Here α denotes cavity modes, while {η_*p*_^α^} and {*z*_α_} are variational parameters. The difference with respect to SC-QED-MP2 is the basis choice. In eq 55, the  operator is in the orthogonalized Löwdin basis, which differs from the dipole basis that diagonalizes (**d**·**ϵ**). In particular, the atomic basis functions are orthogonalized by means of the **S**^–1/2^ matrix, where **S** is the overlap matrix between the AOs.

Furthermore, the total transformation in eq 54 is redundant. In fact, the two transformations commute and *U*_CS_ can be reabsorbed in *U*_LF_ by an appropriate shift of the {η_*p*_^α^} parameters. For this reason, in the remaining of this Section we neglect the {*z*_α_} parameters. Using a generic basis seems advantageous for developing a multimode *ab initio* polaritonic theory. However, there are physical reasons that keep us relying on the dipole basis. Not only in the dipole basis is the wave function exact in the infinite coupling limit, but not using it may lead to non size-intensive molecular orbitals. In fact, from a preliminary theoretical investigation, the single-mode DSE contribution to the Fock matrix elements is
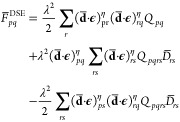
57

For a bipartite system where the subsystems A and B are far apart, the *r* and *s* summation in the Coulomb term (second line in eq 57) is over both A and B orbitals. This implies that the Fock matrix, as in QED-MP2, is not size-intensive because of the contributions from system B to the  elements. The theoretical argument extends straightforwardly to the multimode case.

## 3 Results and Discussions

In this Section, we assess the performance of the QED-MP2, QED(np-HF)-MP2, LF-MP2 and the developed SC-QED-MP2 methods. We focus on cavity coupling and frequency dispersions, intermolecular potential energy curves for different kinds of interactions and last orientational effects of the polarization vector with respect to the molecular system. We use the QED-CCSD^28^ as the benchmark for the perturbative results. Specifically, this coupled cluster theory is obtained using QED-HF as a reference wave function and is expected to capture more correlation energy than SC-QED-MP2 due to the inclusion of unlinked excitations entering the many-body exponential cluster operator. For this reason, although the SC-QED-MP2 method is based on a different reference wave function, the comparison is justified as we focus on electron-photon correlation effects. The SC version of QED-CC is currently under development and we expect it to capture more correlation than its QED-HF counterpart. The QED-MP2, QED(np-HF)-MP2 and SC-QED-MP2 methods have been implemented in a development version of the  program.^58^ The LF-MP2 calculations have been performed using the Polar program,^59^ which is supported with some routines from the PySCF package.^60^ All calculations have been performed using the aug-cc-pVDZ basis set.^61,62^ The molecular structures have been optimized using the ORCA software^63^ with DFT-B3LYP functional and using a def2-SVP basis set. We point out that to compare the Møller–Plesset perturbative methods with the QED-CCSD theory, one should focus on the qualitative trend of the curves rather than total energies. Perturbative methods, as well as coupled cluster approaches, are nonvariational and lower energies are not necessarily indicative of better performance. For the results obtained with the SC-QED-MP2 approach, the infinite summation in the photonic space in eq 49 is truncated when the contributions are smaller than 10^–12^ a.u. In the LF-MP2 calculations the truncation is made after considering 17 photons in the photonic space, which is more than enough to converge the results.

### 3.1 Cavity Coupling and Frequency Dispersions

**Figure 1:**
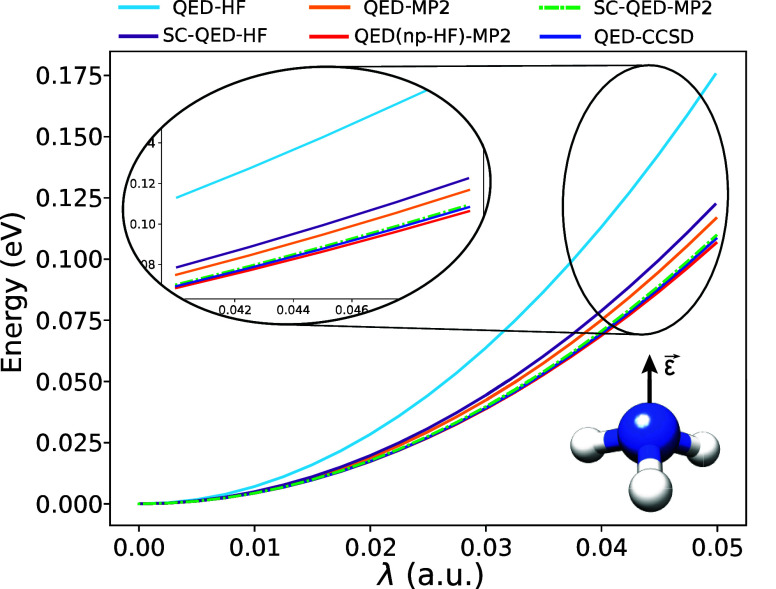
Coupling dispersions for an ammonia molecule. The cavity frequency is set to ω = 8.16 eV, while the polarization ϵ is along the *C*_3_ axis. For realistic coupling values, λ ≤ 0.05 a.u., all the methods show the same increasing trend. For SC-QED-MP2, the inclusion of electron-photon correlation at the mean-field level becomes important for larger couplings.

In Figure 1, we show the energy dispersions of an ammonia molecule in an optical cavity with a frequency of ω = 8.16 eV as a function of the light-matter coupling parameter λ. The cavity polarization is aligned along the *C*_3_ symmetry axis of the molecule. The λ = 0 a.u. energy is set to zero for all the methods. All the presented methodologies capture the qualitative effect of the field, i.e. the energy increases with increasing light-matter coupling. The mean-field approaches, i.e. the QED-HF and SC-QED-HF, overestimate this trend, which is decreased by including more electron-photon correlation. However, we observe that the SC-QED-HF approach performs better by capturing some electron-photon correlation already at the mean-field level.^45,46^ Within the Møller–Plesset methods, QED-MP2 performs worse than the others, overestimating the trend of QED-CCSD and lying close to SC-QED-HF at higher couplings. This is not surprising considering the ill-defined molecular orbitals of the reference QED-HF. The QED(npHF)-MP2 and SC-QED-MP2 methods perform well, following for all coupling values the QED-CCSD trend. However, as discussed in Section 3, we expect that for very strong values of the light-matter coupling the QED(np-HF)-MP2 should exhibit a decrease in accuracy. This effect is displayed in the zoom panel on the left where, at higher couplings, the SC-QED-MP2 becomes the most accurate methodology. This observation is in agreement with the fact that the reference SC-QED-HF wave function becomes exact in the infinite coupling limit. However, we point out that realistic λ values for strongly coupled systems are smaller than 0.05a.u. (corresponding to a quantization volume of around 1 nm^3^).

**Figure 2:**
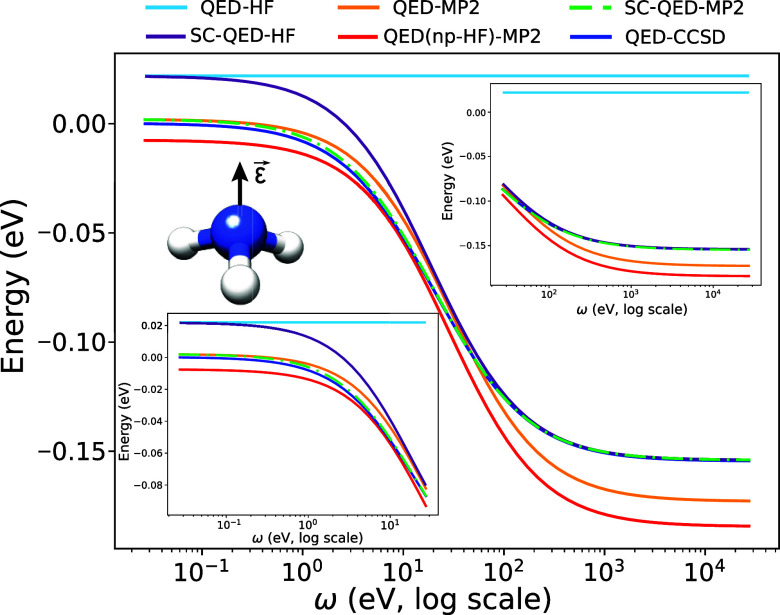
Frequency dispersions for an ammonia molecule. The cavity light-matter coupling is set to λ = 0.05 a.u., while the polarization **ϵ** is along the *C*_3_ axis. The SC-QED-MP2 approach reproduces well the QED-CCSD trend for the whole range of ω.

In Figure 2, we plot the cavity frequency energy dispersions for the same system with the light-matter coupling set to λ = 0.05 a.u. The offset is chosen in order to unbias the comparisons with respect to the electron–electron correlation. To this end, the QED-HF and SC-QED-HF curves are shifted by the electron–electron correlation captured by CCSD outside the cavity. On the other hand, for QED-MP2, QED(np-HF)-MP2 and SC-QED-MP2 we shift the curves by the difference of the electron–electron correlation between MP2 and CCSD outside the cavity. Finally, the zero energy point is equal to the QED-CCSD results at low frequencies. One of the main strengths of SC-QED-HF is its ability to exhibit a qualitatively correct frequency dispersion of the energy, even at the mean-field level.^45,46^ The SC-QED-HF curve matches the QED-CCSD dispersion at extremely high frequencies. In fact, in that range of ω, light and matter are effectively decoupled. At small cavity frequencies, the SC-QED-HF curve overestimates the QED-CCSD results because of the additional electron-photon correlation captured by coupled cluster. In contrast, the QED-HF method does not display any correlation at all and the energy remains constant. All the Møller–Plesset methodologies display the correct dispersion trend. However, the QED-MP2 and QED(np-HF)-MP2 methods perform worse compared to SC-QED-MP2, which matches the QED-CCSD results for the whole frequency range. We claim that this happens because the electron-photon correlation is captured only perturbatively by QED-MP2 and QED(np-HF)-MP2. The SC-QED-MP2, on the other hand, relies on a cavity-consistent set of molecular orbitals and the electron-photon correlation is already naturally included in the zeroth-order Hamiltonian.

### 3.2 Intermolecular Interactions

**Figure 3:**
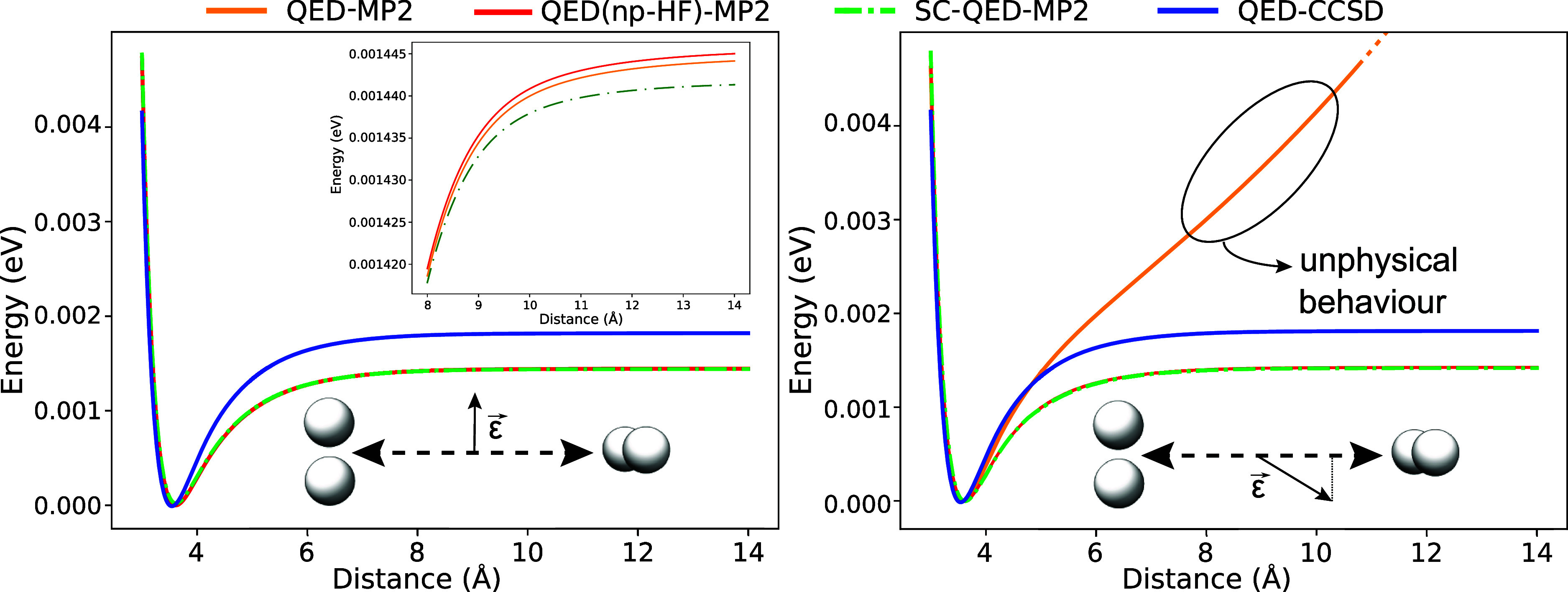
Dissociation curves for two H_2_ molecules in an optical cavity with frequency and light-matter coupling set to ω = 27.2 eV and λ = 0.01 a.u. On the left the polarization ϵ is orthogonal to the displacement direction, while on the right it has a component . When the polarization has a component along the displacement direction the QED-MP2 method displays an unphysical behavior in the long-range regime.

Long-range effects become significant when intermolecular interactions are considered, for example in the case of the van der Waals interaction between two hydrogen molecules shown in Figure 3. In particular, we plot the dissociation potential energy curves of the Møller–Plesset approaches. All the curves are shifted such that the minima are set to zero. The cavity frequency and the light-matter coupling are set to ω = 27.2 eV and λ = 0.01 a.u. On the left we show the results for a polarization along to the *z* axis (orthogonal to the displacement direction). On the right, instead, we plot the results for a field polarization of . For the first polarization we notice that all the methods provide a good description around the equilibrium distance. The Møller–Plesset methods overestimate the attractive part of the potential ∝ – 1/*r*^6^. From the second polarization on the right, the QED(npHF)-MP2 and SC-QED-MP2 perform in a similar way, while the QED-MP2 displays an unphysical behavior in the long-range regime. For symmetry arguments, this behavior has to be due to the polarization component along the displacement direction. We stress that the system is not charged. For this reason the issue does not emerge from the origin dependent molecular orbitals for charged systems. The problem stems from the non size-intensivity of the zeroth-order Hamiltonian which is based on ill-defined orbital energies for separated systems.

**Figure 4:**
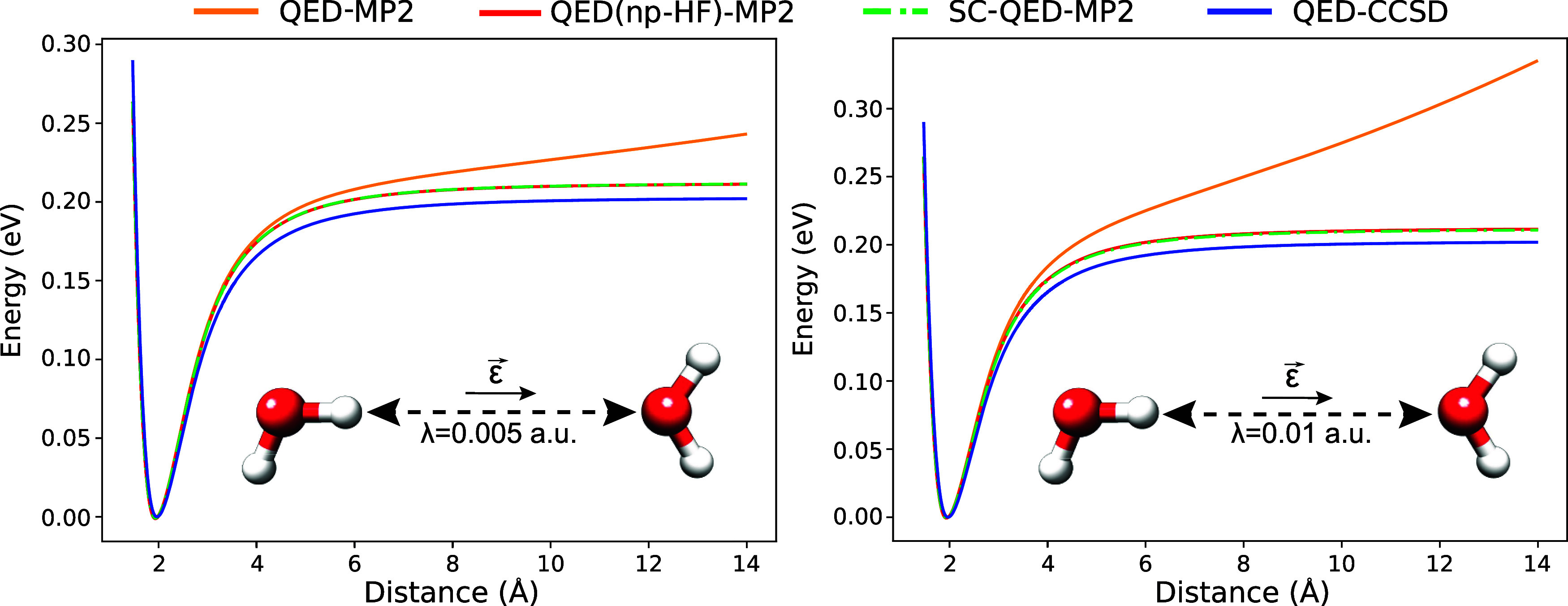
Dissociation curves for two water molecules in a hydrogen bonding geometry inside a cavity. The frequency is set to ω = 8.16 eV, while the polarization **ϵ** is along to the displacement direction. The light-matter coupling is set to λ = 0.005 a.u. on the left and λ = 0.01 a.u. on the right. The unphysical behavior displayed by QED-MP2 is enhanced at higher couplings.

In Figure 4, we investigate the behavior of the Møller–Plesset approaches for a hydrogen-bonded geometry of the water dimer. The leading term of the intermolecular interaction is given by the dipole–dipole interaction, however, the charge-transfer component along the hydrogen bridge is known to be non-negligible.^64^ The cavity frequency is set to ω = 8.16 eV, while the light-matter coupling is set to λ = 0.005 a.u. in the left plot and λ = 0.01 a.u. in the right one. For both the plots the polarization vector is along the *y* axis (the displacement direction). The QED(np-HF)-MP2 and SC-QED-MP2 reproduce qualitatively well the potential curve but underestimate the binding energy, contrary to what is observed for the van der Waals interaction in Figure 3. With the polarization along the displacement direction, the QED-MP2 approach displays again an unphysical behavior due to the ill-defined molecular orbitals of the reference QED-HF. Comparing the plots at different couplings we can see that the issue is enhanced with increasing the light-matter coupling λ. This observation is consistent with the λ^2^-scaling of the non size-intensive terms of the Fock matrix elements in eq 13.

**Figure 5:**
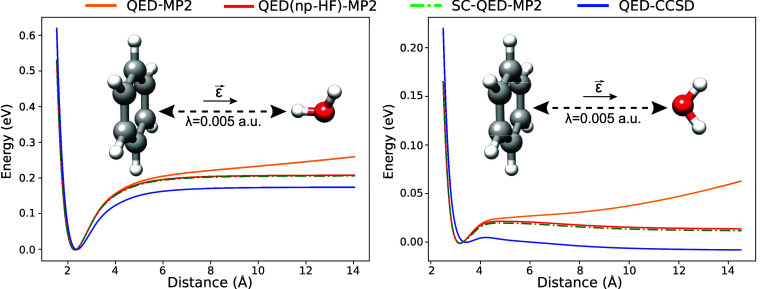
Dissociation curves inside a cavity for a benzene and a water molecule in two different geometries. For both cases the frequency is set to ω = 2.27 eV, while light-matter the coupling is set to λ = 0.005 a.u. The polarization **ϵ** is along to the displacement direction. The unphysical behavior displayed by QED-MP2 changes an unbounded intermolecular interaction into a bounded one (see plot on right).

In Figure 5, we show the behavior of the perturbative approaches for a dipole–induced dipole system composed by a benzene and a water molecule. The polarization vector **ϵ** is again along the displacement direction, while the light-matter coupling and cavity frequency are set to λ = 0.005 a.u. and ω = 2.27 eV. On the left, the system is bonded because one of the water hydrogens points toward the benzene. On the right, instead, the system shows a metastable minima because the oxygen of the water molecule is pointing toward the ring. This is due to the repulsive interaction between the π electrons cloud of the benzene and the lone pairs on the oxygen atom. Yet again, the trend of the Møller–Plesset approaches is the same, with the QED-MP2 displaying an unphysical behavior. However, for the metastable system on the right, this issue is even more troublesome because a physically unbounded system is turned into a bounded one.

**Figure 6:**
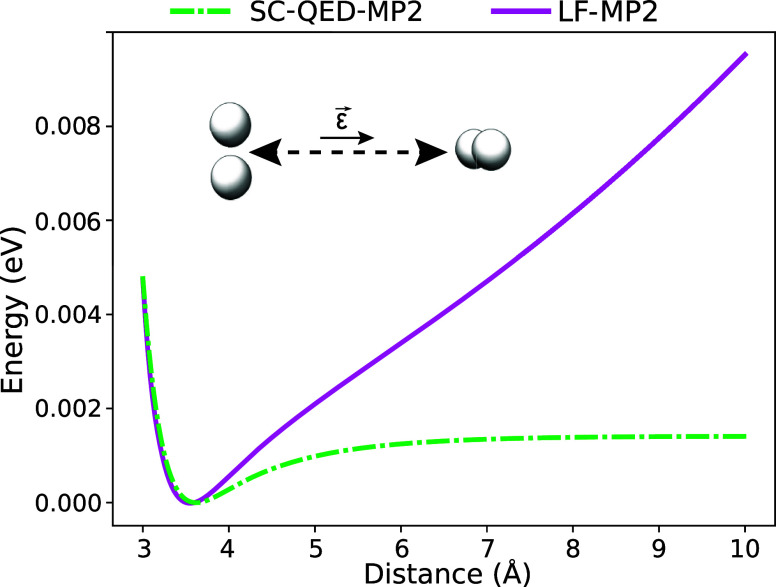
Comparison between SC-QED-MP2 and LF-MP2 dissociation curves for two H_2_ molecules in an optical cavity with frequency and light-matter coupling set to ω = 2.72 eV and λ = 0.01 a.u. The polarization **ϵ** is along the displacement direction. The LF-MP2 method displays the same unphysical behavior as QED-MP2.

From the results shown, is clear that SC-QED-MP2 and QED(npHF)-MP2 are well behaved and capture the different kinds of intermolecular interactions. Using a wave function parametrization similar to SC-QED-HF, but in another basis, is not sufficient to obtain the similar accuracy. In Figure 6, we show the comparison between SC-QED-MP2 and LF-MP2 for the two hydrogen van der Waals system. The cavity frequency and the light-matter coupling are set to ω = 2.72 eV and λ = 0.01 a.u., while the polarization vector **ϵ** is along the displacement direction. The LF-MP2 curve behaves like the one for QED-MP2 by displaying the same unphysical diverging trend in the long-range regime. This behavior could be due to the non size-intensivity of the Fock matrix in a basis that differs from the dipole basis as shown in eq 57. An alternative explanation for this behavior has an algorithmic nature. The existence of a bifurcation point in the parameter space that is not accurately treated in the optimization procedure can lead to the convergence to another solution different from the ground state. Further investigations are necessary, but these observations suggest that the choice of basis is crucial in ab initio polaritonic models.

### 3.3 Polarization Orientational Effects

**Figure 7:**
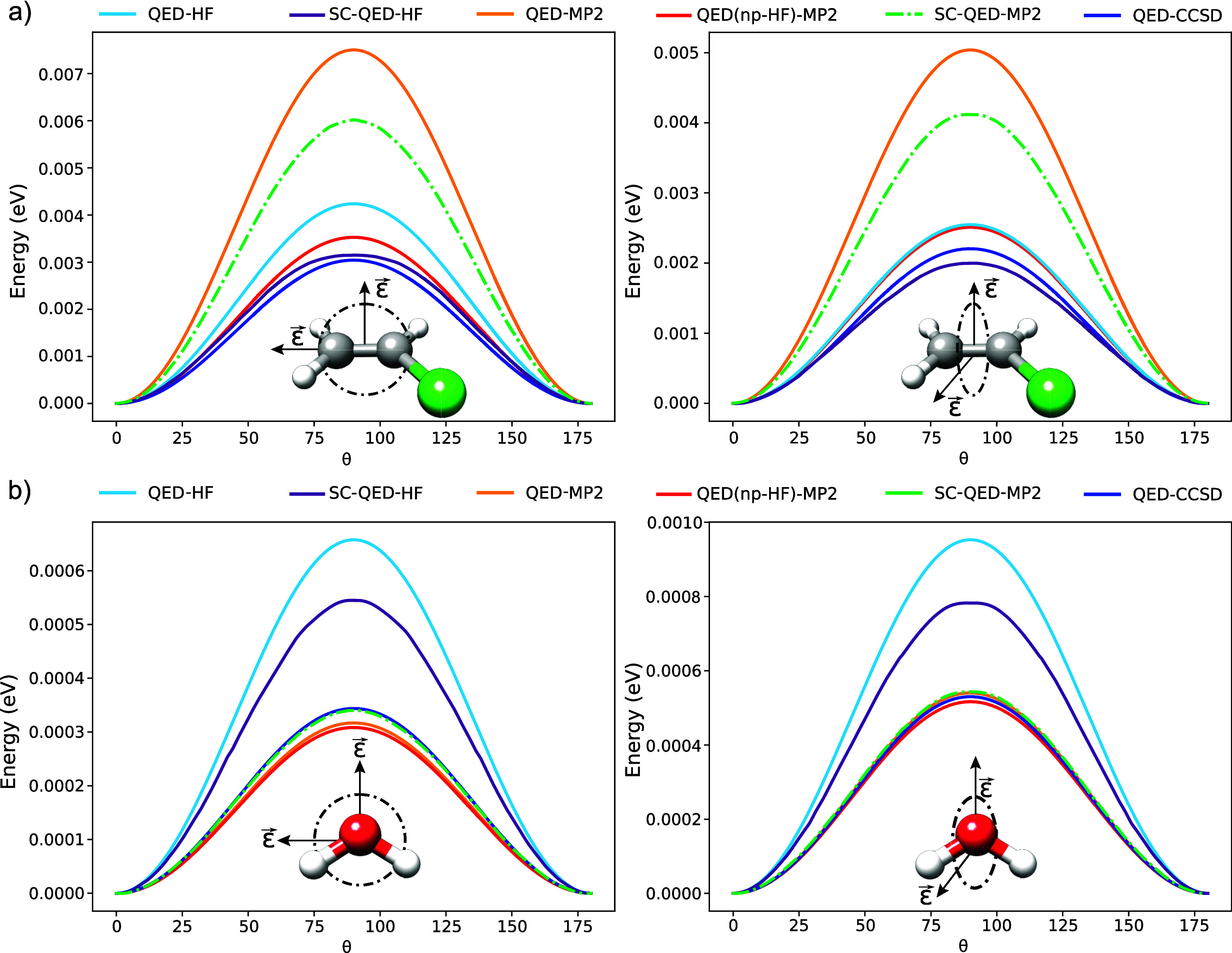
Field polarization ϵ orientational effects on chloroethylene (a) and water (b) inside an optical cavity with frequency and the light-matter coupling set to ω = 2.72 eV and λ = 0.01 a.u. For both molecules, two orthogonal rotations of the field polarization are shown.

In Figure 7, we investigate the orientational effects of the field polarization **ϵ** for chloroethylene (a) and water (b). For both molecular systems we perform two orthogonal rotations of the polarization vector ϵ (left and right plots). The cavity frequency and the light-matter coupling are set to ω = 2.72 eV and λ = 0.01 a.u. The offset of the curves is chosen such that they all start and end at zero energy. In all the four cases we observe that the θ-dispersions reach a maximum when the polarization lies in the molecular plane. The closer the polarization vector is to the molecular plane, the orientational effects are described less accurately by the mean-field and perturbative approaches. For chloroethylene (a) the SC-QED-HF performs the best, while all the other approaches overestimate the orientational effects. Among the perturbative methods, the QED(np-HF)-MP2 outperforms the others. For water (b), instead, we observe that the perturbative approaches perform collectively better with respect to the mean-field methods. In particular, when the rotation is parallel to the *C*_2_ symmetry axis, the SC-QED-MP2 performs best. In the orthogonal case, instead, the QED-MP2 and QED(np-HF)-MP2 are closer to the QED-CCSD curve. These orientational effects are purely polaritonic and come from the interplay between DSE contributions and electron-photon correlation. The DSE gives a positive contribution to the energy reaching its maximum when the cavity polarization and the largest polarizability principal axis are aligned. On the other hand, the electron-photon correlation has a negative contribution to the energy such that the overall observed behavior results from cancellation of these two effects.

## 4 Conclusions

In this study, building upon the work of Bauer et al.,^44^ we have developed of a Møller–Plesset perturbation theory based on the reliable SC-QED-HF polaritonic molecular orbitals.^45,46^ Our analysis reveals that employing a fully consistent molecular orbital framework for the zeroth-order Hamiltonian is critical to effectively capture the cavity-induced electron-photon correlation effects. For instance, the cavity coupling and frequency dispersions are well reproduced by the SC-QED-MP2 approach which includes some correlation effects at the mean-field level. On the other hand, the QED-MP2 and QED(np-HF)-MP2 methods capture the electron-photon correlation only perturbatively, and higher orders in perturbation theory may be needed in order to obtain higher accuracy. However, Møller–Plesset perturbation theory is not guaranteed to converge and further investigations of the converge properties in the polaritonic Hilbert space  may provide interesting numerical insights.^65−67^ The use of a correct molecular orbital theory is crucial in order to avoid unphysical behavior, such as the ones displayed by QED-MP2 and LF-MP2 in the long-range regime of intermolecular interactions. We also point out that the generalization to an *ab initio* multimode QED Hamiltonian is less trivial than just having a diagonal transformation for each mode. Care must be taken in order to obtain a multimode molecular orbital theory. Efforts in this direction are currently under way. This work paves the way for the development of more accurate perturbative approaches, such as QED versions of CC2 and CC3. Similarly, active space methodologies can only be extended to QED environments for fully consistent molecular orbital theories. Continuous advancements in experimental setups to achieve larger coupling strengths may advocate for using SC-QED-MP2 in forthcoming studies.

## Data Availability

The development version of the  program^58^ used to perform the calculations shown in this work is available from the corresponding author upon reasonable request. The data of this work are available at https://zenodo.org/records/14639372.

## Supporting Information Available

Development of general QED Møller–Plesset perturbation theory for multiple set of bosons and modes. Derivation of strong coupling QED Møller–Plesset perturbation theory (PDF)
