# Supplementary material for: Strong Coupling Møller–Plesset Perturbation Theory
Source: J Chem Theory Comput. 2025 Mar 31;21(8):3981–92. doi: 10.1021/acs.jctc.5c00055 (PMC12020365; doi:10.1021/acs.jctc.5c00055)
Supplement: Supplementary file 1 — ct5c00055_si_001.pdf [file ct5c00055_si_001.pdf]

Supporting information to: Strong coupling Møller-Plesset perturbation theory

Yassir El Moutaoukal,[†,‡] Rosario R. Riso,[†,‡] Matteo Castagnola,[†] Enrico Ronca,[¶] and Henrik Koch[*,†]

†*Department of Chemistry, Norwegian University of Science and Technology, 7491 Trondheim, Norway*

‡*These authors contributed equally to this work*

¶*Department of Chemistry, Biology and Biotechnology, University of Perugia, Via Elce di Sotto, 8, 06123, Perugia, Italy*

E-mail: henrik.koch@ntnu.no

S1. QED Møller-Plesset perturbation theory

Consider an *ab initio* QED Hamiltonian H able to model a molecular system interacting with a set $\{\alpha\}$ of non-interacting bosons.[1] This many-body Hamiltonian can be split in three terms

$$H = H_{mol} + \sum_{\alpha} H_{\alpha} + H_{int}, \tag{SE1}$$

where H_{mol} is the molecular Hamiltonian, H_{α} is the α-boson Hamiltonian and lastly H_{int} is the interaction between the molecular system and the bosonic fields. Working in the Born-Oppenheimer approximation, the molecular Hamiltonian H_{mol} reduces to the electronic Hamiltonian

$$H_{el} = \sum_{pq} h_{pq} E_{pq} + \frac{1}{2} \sum_{pqrs} g_{pqrs} e_{pqrs}, \tag{SE2}$$

where h_{pq} and g_{pqrs} are the one and two electron integrals. The second quantization formalism is adopted, so

$$\begin{aligned} E_{pq} &= \sum_{\sigma} a^{\dagger}_{p\sigma} a_{q\sigma} \\ e_{pqrs} &= E_{pq}E_{rs} - \delta_{rq}E_{ps}, \end{aligned} \tag{SE3}$$

with $a^{\dagger}_{p\sigma}$ and $a_{p\sigma}$ respectively creating and annihilating an electron in the orbital p with spin σ. On the other hand, each field Hamiltonian H_{α} is written as a collection of quantum harmonic oscillators

$$H_{\alpha} = \sum_{k_{\alpha}} \omega_{k_{\alpha}} b^{\dagger}_{k_{\alpha}} b_{k_{\alpha}}, \tag{SE4}$$

where k runs over boson modes and $b_{k,\alpha}$, $b^{\dagger}_{k,\alpha}$ do annihilate, create bosons of frequency $\omega_{k,\alpha}$. These harmonic oscillators are generally coupled linearly with the electronic degrees of freedom

$$H_{int} = \sum_{k_{\alpha}} \sum_{pq} g^{k_{\alpha}}_{pq} E_{pq} (b_{k_{\alpha}} + b^{\dagger}_{k_{\alpha}}), \tag{SE5}$$

where $g^{k_{\alpha}}_{pq}$ are coupling constants that can be in any order of accuracy in the multipolar expansion of the interaction.

This Hamiltonian

$$H = H_{el} + \sum_{k_{\alpha}} \omega_{k_{\alpha}} b^{\dagger}_{k_{\alpha}} b_{k_{\alpha}} + \sum_{k_{\alpha}} \sum_{pq} g^{k_{\alpha}}_{pq} E_{pq} (b_{k_{\alpha}} + b^{\dagger}_{k_{\alpha}}) \tag{SE6}$$

is defined in a space

$$\mathcal{H} = \mathcal{H}_{el} \bigotimes_{\alpha} \mathcal{H}_{\alpha}, \tag{SE7}$$

where $\mathcal{H}_{el}$ is the electronic Hilbert space and $\mathcal{H}_{\alpha}$ is the α-boson Hilbert space. This tensor space is spanned by the states

$$\{|\mu\rangle \bigotimes_{\alpha} U^{coh}_{\alpha} |n_{1_{\alpha}}, ..., n_{k_{\alpha}}, ...\rangle\} \tag{SE8}$$

where $|\mu\rangle$ is an electronic occupation number state (ONS) and $|n_{1_\alpha}, ..., n_{k_\alpha}, ...\rangle$ is an α-bosonic ONS. For the electronic ONSs $|\mu\rangle$, the quasiparticle formalism is adopted by considering as a reference the Hartree-Fock state

$$|\mathrm{HF}\rangle = \prod_{i,\sigma}^{n_{occ}} a_{i\sigma}^{\dagger} |vac\rangle \,, \tag{SE9}$$

where the low-lying molecular orbitals of the electronic vacuum $|vac\rangle$ are occupied. So, the $|\mu\rangle$ states are defined as excitations of the reference $|\mathrm{HF}\rangle$

$$|\mu\rangle = \tau_\mu |\mathrm{HF}\rangle \,, \tag{SE10}$$

where τ_μ is an excitation operator and considering $\tau_0 = I$, the identity. For the α-boson ONSs, we consider as a reference the vacuum

$$|0_\alpha\rangle = |0_{1_\alpha}, ..., 0_{k_\alpha}, ...\rangle \,. \tag{SE11}$$

So, the $|n_{1_\alpha}, ..., n_{k_\alpha}, ...\rangle$ are defined as excitations on this vacuum

$$|n_{1_\alpha}, ..., n_{k_\alpha}, ...\rangle = \prod_{k_\alpha} \frac{1}{\sqrt{n_{k_\alpha}!}} (b_{k_\alpha}^{\dagger})^{n_{k_\alpha}} |0_\alpha\rangle \,, \tag{SE12}$$

where the set $\{n_{\kappa_\alpha}\}$ are the occupation numbers of the α-boson modes. The U_α^{coh} in eq. (SE8) are coherent-states transformations of the form

$$U_\alpha^{coh} = \prod_{k_\alpha} \exp\Big(-z_{k_\alpha}(b_{k_\alpha} - b_{k_\alpha}^{\dagger})\Big), \tag{SE13}$$

where z_{k_α} is a coherent-state parameters. We can change the quantum picture by passing in

the coherent-state basis for each boson and applying all the U_α^{coh} to the Hamiltonian

$$H_{coh} = \prod_\alpha U_\alpha^{coh\ \dagger}\ H\ U_\alpha^{coh}. \tag{SE14}$$

In this picture, the Hamiltonian is defined in a space spanned by the states

$$\{|\mu\rangle \bigotimes_\alpha |n_{1_\alpha}, ..., n_{k_\alpha}, ...\rangle \ \equiv |\mu, \{n_{k_\alpha}\}\rangle\}. \tag{SE15}$$

Due to the correlation between the particles of the many-body system, the exact eigenstates of H_{coh} are complicated to model and most likely unknown in a finite order expansion of the basis in eq. (SE15). For this reason we can rely on Rayleigh-Shrödinger perturbation theory and split H_{coh} in a zeroth-order solvable part $H_{coh}^{(0)}$ and a fluctuation potential V where all the correlation effects do reside

$$H_{coh} = H_{coh}^{(0)} + \gamma V. \tag{SE16}$$

The γ parameter is introduced in order to keep track of the perturbation orders of the expansion throughout the derivation. Following the Møller-Plesset (MP) scheme for electronic structure theory,[2] in the zeroth-order Hamiltonian we can consider the Fock operator coming from a mean-field treatment of the correlation effects

$$F_{coh} = \sum_{pq} F_{pq}^{coh} E_{pq}, \tag{SE17}$$

where F_{pq}^{coh} are Fock matrix elements. Due to the presence of the bosons in the overall system, it is wise to add the harmonic oscillators in $H_{coh}^{(0)}$ as well

$$H_{coh}^{(0)} = \sum_{pq} F_{pq}^{coh} E_{pq} + \sum_{k_\alpha} \omega_{k_\alpha} b_{k_\alpha}^\dagger b_{k_\alpha}. \tag{SE18}$$

The states in eq. (SE15) are eigenfunctions of (SE18)

$$H^{(0)}_{coh}\left|\mu,\{n_{k_\alpha}\}\right\rangle^{(0)} = E^{(0)}_{\mu,\{n_{k_\alpha}\}}\left|\mu,\{n_{k_\alpha}\}\right\rangle^{(0)} \tag{SE19}$$

with eigenvalues

$$E^{(0)}_{\mu,\{n_{k_\alpha}\}} = E^{(0)}_{\mathrm{HF},\{0_{k_\alpha}\}} + \epsilon_\mu + \sum_{k_\alpha} n_{k_\alpha}\omega_{k_\alpha}, \tag{SE20}$$

where $E_{\mathrm{HF},\{0_{k_\alpha}\}}$ is the ground state zeroth order energy and ϵ_μ is the excitation energy between the the electronic $|\mathrm{HF}\rangle$ and $|\mu\rangle$ Slater determinants. To obtain eq. (SE20) we made use of the diagonal form of the Fock matrix in the canonical basis. The eigenstates $\left|\mu,\{n_{k_\alpha}\}\right\rangle^{(0)}$ are the ones spanning the coupled electron-bosons Hilbert space where the coherent-state transformed Hamilotnian is defined (eq. (SE15)). The fluctuation potential V is straightforwardly defined as

$$V = H_{coh} - H^{(0)}_{coh}\ . \tag{SE21}$$

In order to see how the states in eq. (SE15) are affected by the presence of the perturbation V, we expand them perturbatively in γ

$$\left|\psi_{\mu,\{n_{k_\alpha}\}}\right\rangle = \left|\mu,\{n_{k_\alpha}\}\right\rangle^{(0)} + \gamma\left|\mu,\{n_{k_\alpha}\}\right\rangle^{(1)} + \gamma^2\left|\mu,\{n_{k_\alpha}\}\right\rangle^{(2)} + ... \tag{SE22}$$

and the same we do for the associated energies

$$E_{\mu,\{n_{k_\alpha}\}} = E^{(0)}_{\mu,\{n_{k_\alpha}\}} + \gamma E^{(1)}_{\mu,\{n_{k_\alpha}\}} + \gamma^2 E^{(2)}_{\mu,\{n_{k_\alpha}\}} + ... \tag{SE23}$$

The terms in zeroth-order with respect to γ are the ones showing in eq. (SE19) and (SE20). Now, we can insert eqs. (SE22) and (SE23) in the Shrödinger equation for the full Hamiltonian

$$H_{coh}\left|\psi_{\mu,\{n_{k_\alpha}\}}\right\rangle = E_{\mu,\{n_{k_\alpha}\}}\left|\psi_{\mu,\{n_{k_\alpha}\}}\right\rangle \tag{SE24}$$

and obtain

$$(H_{coh}^{(0)} + \gamma V)(|\mu, \{n_{k_\alpha}\}\rangle^{(0)} + \gamma |\mu, \{n_{k_\alpha}\}\rangle^{(1)} + \gamma^2 |\mu, \{n_{k_\alpha}\}\rangle^{(2)} + ...) =$$
$$= (E^{(0)}_{\mu,\{n_{k_\alpha}\}} + \gamma E^{(1)}_{\mu,\{n_{k_\alpha}\}} + \gamma^2 E^{(2)}_{\mu,\{n_{k_\alpha}\}} + ...)(|\mu, \{n_{k_\alpha}\}\rangle^{(0)} + \gamma |\mu, \{n_{k_\alpha}\}\rangle^{(1)} + \gamma^2 |\mu, \{n_{k_\alpha}\}\rangle^{(2)} + ...). \tag{SE25}$$

We notice that the equation must hold in each order γ^n of the perturbation expansion:

$$\gamma^0 : \quad H_{coh}^{(0)} |\mu, \{n_{k_\alpha}\}\rangle^{(0)} = E^{(0)}_{\mu,\{n_{k_\alpha}\}} |\mu, \{n_{k_\alpha}\}\rangle^{(0)} \tag{SE26}$$

$$\begin{aligned} \gamma^1 : \quad & H_{coh}^{(0)} |\mu, \{n_{k_\alpha}\}\rangle^{(1)} + V |\mu, \{n_{k_\alpha}\}\rangle^{(0)} = \\ & = E^{(0)}_{\mu,\{n_{k_\alpha}\}} |\mu, \{n_{k_\alpha}\}\rangle^{(1)} + E^{(1)}_{\mu,\{n_{k_\alpha}\}} |\mu, \{n_{k_\alpha}\}\rangle^{(0)} \end{aligned} \tag{SE27}$$

$$\begin{aligned} \gamma^2 : \quad & H_{coh}^{(0)} |\mu, \{n_{k_\alpha}\}\rangle^{(2)} + V |\mu, \{n_{k_\alpha}\}\rangle^{(1)} = \\ & = E^{(0)}_{\mu,\{n_{k_\alpha}\}} |\mu, \{n_{k_\alpha}\}\rangle^{(2)} + E^{(1)}_{\mu,\{n_{k_\alpha}\}} |\mu, \{n_{k_\alpha}\}\rangle^{(1)} + E^{(2)}_{\mu,\{n_{k_\alpha}\}} |\mu, \{n_{k_\alpha}\}\rangle^{(0)} \end{aligned} \tag{SE28}$$

$$...$$

At the zeroth-order in (SE26) we have the Shrödinger equation in (SE19).

For the first-order in (SE27) we can expand $|\mu, \{n_{k_\alpha}\}\rangle^{(1)}$ in the zeroth-order basis

$$|\mu, \{n_{k_\alpha}\}\rangle^{(1)} = \sum_{\nu,\{n_{k_\beta}\}} c^{(1)}_{\mu,\{n_{k_\alpha}\};\ \nu,\{n_{k_\beta}\}} \ |\nu, \{n_{k_\beta}\}\rangle^{(0)} \tag{SE29}$$

and project on ${}^{(0)}\langle\mu, \{n_{k_\alpha}\}|$ to obtain the first-order energy correction

$$E^{(1)}_{\mu,\{n_{k_\alpha}\}} = {}^{(0)}\langle\mu, \{n_{k_\alpha}\}|V\,|\mu, \{n_{k_\alpha}\}\rangle^{(0)}\,. \tag{SE30}$$

On the other hand, if we project on a generic ${}^{(0)}\langle\rho, \{n_{k_\theta}\}|$ different from ${}^{(0)}\langle\mu, \{n_{k_\alpha}\}|$, we obtain the expression for the expansion coefficients in eq. (SE29)

$$c^{(1)}_{\mu,\{n_{k_\alpha}\};\;\nu,\{n_{k_\beta}\}} = -\frac{{}^{(0)}\langle\nu, \{n_{k_\beta}\}|V\,|\mu, \{n_{k_\alpha}\}\rangle^{(0)}}{E^{(0)}_{\nu,\{n_{k_\beta}\}} - E^{(0)}_{\mu,\{n_{k_\alpha}\}}} \quad ; \quad \nu, \{n_{k_\beta}\} \neq \mu, \{n_{k_\alpha}\}. \tag{SE31}$$

To obtain eq. (SE30) and eq. (SE31) we made use of the orthogonality of the zeroth-order basis and of eq. (SE26). So, the first-order correction of the basis eigenstates is

$$|\mu, \{n_{k_\alpha}\}\rangle^{(1)} = |\mu, \{n_{k_\alpha}\}\rangle^{(0)} - \sum_{\nu,\{n_{k_\beta}\}} \frac{{}^{(0)}\langle\nu, \{n_{k_\beta}\}|V\,|\mu, \{n_{k_\alpha}\}\rangle^{(0)}}{E^{(0)}_{\nu,\{n_{k_\beta}\}} - E^{(0)}_{\mu,\{n_{k_\alpha}\}}}\; |\nu, \{n_{k_\beta}\}\rangle^{(0)}\,. \tag{SE32}$$

For the second-order in (SE28) we can again expand $|\mu, \{n_{k_\alpha}\}\rangle^{(2)}$ in the zeroth-order basis

$$|\mu, \{n_{k_\alpha}\}\rangle^{(2)} = \sum_{\nu,\{n_{k_\beta}\}} c^{(2)}_{\mu,\{n_{k_\alpha}\};\;\nu,\{n_{k_\beta}\}}\; |\nu, \{n_{k_\beta}\}\rangle^{(0)} \tag{SE33}$$

and project on ${}^{(0)}\langle\mu, \{n_{k_\alpha}\}|$ to obtain the second-order energy correction

$$E^{(2)}_{\mu,\{n_{k_\alpha}\}} = -\sum_{\nu,\{n_{k_\beta}\}} \frac{{}^{(0)}\langle\nu, \{n_{k_\beta}\}|V\,|\mu, \{n_{k_\alpha}\}\rangle^{(0)}\,{}^{(0)}\langle\mu, \{n_{k_\alpha}\}|V\,|\nu, \{n_{k_\beta}\}\rangle^{(0)}}{E^{(0)}_{\nu,\{n_{k_\beta}\}} - E^{(0)}_{\mu,\{n_{k_\alpha}\}}} \quad ; \quad \nu, \{n_{k_\beta}\} \neq \mu, \{n_{k_\alpha}\}. \tag{SE34}$$

Yet again, with the same technique used before we can determine the second-order correction to the eigenstates.

Iterating the procedure we can obtain the energy and eigenstates corrections in all orders of the Møller-Plesset perturbation hierarchy with the well known $2n + 1$ rule.

S2. Strong coupling QED-MP2

We consider the single-mode Pauli-Fierz Hamiltonian modeling the light-matter interaction between a molecular system and a cavity photon[3]

$$H = H_e + \omega b^\dagger b + \frac{\lambda^2}{2}(\mathbf{d}\cdot\boldsymbol{\epsilon})^2 - \lambda\sqrt{\frac{\omega}{2}}(\mathbf{d}\cdot\boldsymbol{\epsilon})(b^\dagger + b) \tag{SE35}$$

where b and $b^\dagger$ annihilate and create a photon of frequency ω and with polarization $\boldsymbol{\epsilon}$. This Hamiltonian is in the length gauge, with

$$\mathbf{d} = \sum_{pq}\mathbf{d}_{pq}E_{pq} \tag{SE36}$$

being the molecular dipole operator. The λ parameter represents the light-matter coupling strength. For the ground-state wave function parametrization we consider the SC-QED-HF Ansatz[4,5]

$$|\psi\rangle = \exp\left(-\frac{\lambda}{\sqrt{2\omega}}\sum_p \eta_p \tilde{E}_{pp}(b - b^\dagger)\right)|\mathrm{HF}, 0\rangle\,, \tag{SE37}$$

where the electrons are dressed with cavity photons by means of the orbital specific coherent-state transformation

$$U_{\mathrm{SC}} = \exp\left(-\frac{\lambda}{\sqrt{2\omega}}\sum_p \eta_p \tilde{E}_{pp}(b - b^\dagger)\right). \tag{SE38}$$

The tilde $\sim$ symbol denotes integrals and operators in the basis that diagonalize $(\mathbf{d}\cdot\boldsymbol{\epsilon})$:

$$\sum_{rs} C_{rp}(\mathbf{d}\cdot\boldsymbol{\epsilon})_{rs}C_{sq} = (\tilde{\mathbf{d}}\cdot\boldsymbol{\epsilon})_{pp}\delta_{pq}, \tag{SE39}$$

where $\mathbf{C}$ is an orthonormal rotation matrix connecting molecular and dipole orbitals

$$\mathbf{d} = \sum_p \tilde{\mathbf{d}}_{pp}\tilde{E}_{pp}. \tag{SE40}$$

Now we apply the QED Møller-Plesset perturbation theory developed in the previous section to the Hamiltonian (SE35) and the wave function (SE37). Proceeding, we first change the quantum picture by SC-transforming the Pauli-Fierz Hamiltonian

$$\begin{aligned} H_{\mathrm{SC}} &= U_{\mathrm{SC}}^{\dagger}\ H\ U_{\mathrm{SC}} \\ &= \sum_{pq} \tilde{h}_{pq}^{\mathrm{SC}} Y_{pq} \tilde{E}_{pq} + \frac{1}{2}\sum_{pqrs} \tilde{g}_{pqrs}^{\mathrm{SC}} Y_{pqrs} \tilde{e}_{pqrs} \\ &+ \omega b^{\dagger} b - \lambda\sqrt{\frac{\omega}{2}} \sum_{p} ((\tilde{\mathbf{d}}\cdot\boldsymbol{\epsilon})_{pp} - \eta_p)\tilde{E}_{pp}(b + b^{\dagger}), \end{aligned} \tag{SE41}$$

where the redefined SC one and two electron integrals are

$$\tilde{h}_{pq}^{\mathrm{SC}} = \tilde{h}_{pq} + \frac{\lambda^2}{2}((\tilde{\mathbf{d}}\cdot\boldsymbol{\epsilon})_{pp} - \eta_p)^2\delta_{pq} \tag{SE42}$$

$$\tilde{g}_{pqrs}^{\mathrm{SC}} = \tilde{g}_{pqrs} + \lambda^2((\tilde{\mathbf{d}}\cdot\boldsymbol{\epsilon})_{pp} - \eta_p)((\tilde{\mathbf{d}}\cdot\boldsymbol{\epsilon})_{rr} - \eta_r)\delta_{pq}\delta_{rs}, \tag{SE43}$$

while the Y_{pq} and Y_{pqrs} photonic operators are defined as follows

$$Y_{pq} = \exp\left(\frac{\lambda}{\sqrt{2\omega}}(\eta_p - \eta_q)(b - b^{\dagger})\right) \tag{SE44}$$

$$Y_{pqrs} = \exp\left(\frac{\lambda}{\sqrt{2\omega}}(\eta_p - \eta_q + \eta_r - \eta_s)(b - b^{\dagger})\right). \tag{SE45}$$

The Hamiltonian in eq. (SE41) is defined in a space spanned by the states

$$\{|\mu, n\rangle\} \tag{SE46}$$

with $|\mathrm{HF}, 0\rangle$ being the optimized ground state. We define as the SC zeroth-order Hamiltonian

$$H_{\mathrm{SC}}^{(0)} = \sum_{pq} F_{pq}^{\mathrm{SC}} E_{pq} + \omega b^{\dagger} b, \tag{SE47}$$

where the SC-Fock matrix elements in the dipole basis read

$$\tilde{F}^{\text{SC}}_{pq} = \tilde{h}^{\text{SC}}_{pq} Q_{pq} + \frac{1}{2}\sum_{rs}(2\tilde{g}^{\text{SC}}_{pqrs} - \tilde{g}^{\text{SC}}_{psrq}) Q_{pqrs} \tilde{D}_{rs}, \tag{SE48}$$

$\tilde{D}_{pq}$ are elements of the one electron density matrix (in the dipole basis as well)

$$\tilde{D}_{pq} = \langle \text{HF} | \tilde{E}_{pq} | \text{HF} \rangle \tag{SE49}$$

and the Gaussian factors carrying the ω-correlation are

$$Q_{pq} = \langle Y_{pq} \rangle_0 = \exp\left(-\frac{\lambda^2}{4\omega} (\eta_p - \eta_q)^2 \right) \tag{SE50}$$

$$Q_{pqrs} = \langle Y_{pqrs} \rangle_0 = \exp\left(-\frac{\lambda^2}{4\omega} (\eta_p - \eta_q + \eta_r - \eta_s)^2 \right). \tag{SE51}$$

The Fock matrix elements in eqs. (SE47) and (SE48) are connected by

$$\sum_{rs} C_{rp} F_{rs} C_{sq} = \tilde{F}_{pq}, \tag{SE52}$$

where the canonical to dipole basis transformation $\mathbf{C}$ is defined in eq. (SE39).

The states in eq. (SE46) are eigenfunctions of (SE47)

$$H^{(0)}_{\text{SC}} |\mu, n\rangle^{(0)} = E^{(0)}_{\mu n} |\mu, n\rangle^{(0)} \tag{SE53}$$

with eigenvalues

$$E^{(0)}_{\mu n} = E^{(0)}_{\text{HF},0} + \epsilon_\mu + n\omega. \tag{SE54}$$

As discussed in the previous section, the states $|\mu, n\rangle^{(0)}$ are the ones spanning the light-matter Hilbert space where the SC-trasformed Pauli-Fierz Hamiltonian is defined (eq. (SE46)). For this reason, from now on, we drop the zeroth order index for the eigenstates. For $\mu = 0$ the electronic excitation is trivially $\epsilon_0 = 0\,\text{a.u.}$ On the other hand, for excited determinants, we

have for singles ($\mu \in S$), doubles ($\mu \in D$) and so on

$$\epsilon_\mu = \epsilon_a - \epsilon_i, \quad \text{for} \quad |\mu\rangle = E_{ai}\,|\text{HF}\rangle \tag{SE55}$$

$$\epsilon_\mu = \epsilon_a - \epsilon_i + \epsilon_b - \epsilon_j, \quad \text{for} \quad |\mu\rangle = E_{bj}E_{ai}\,|\text{HF}\rangle \tag{SE56}$$

$$...$$

where $i, j, ...$ and $a, b, ...$ label respectively occupied and virtual orbitals in the $|\text{HF}\rangle$ state. Then, the perturbation $V = H_{\text{SC}} - H_{\text{SC}}^{(0)}$ reads

$$\begin{aligned} V = &\sum_{pq} \tilde{h}^{\text{SC}}_{pq}(Y_{pq} - Q_{pq})\tilde{E}_{pq} + \frac{1}{2}\sum_{pqrs} \tilde{g}^{\text{SC}}_{pqrs} Y_{pqrs}\tilde{e}_{pqrs} \\ &- \frac{1}{2}\sum_{pqrs}(2\tilde{g}^{\text{SC}}_{pqrs} - \tilde{g}^{\text{SC}}_{psrq})Q_{pqrs}\tilde{D}_{rs}\tilde{E}_{pq} \\ &- \lambda\sqrt{\frac{\omega}{2}}\sum_{p}((\tilde{\mathbf{d}}\cdot\boldsymbol{\epsilon})_{pp} - \eta_p)\tilde{E}_{pp}(b + b^\dagger). \end{aligned} \tag{SE57}$$

The sum of the zeroth and first-order energy for every state in (SE46) corresponds to the energy associated to that state calculated at the mean-field level of theory. The first correction to these energies happen to be in the second order of the QED Møller-Plesset perturbation theory. With the focus on building up correlation on top of the SC-QED-HF ground-state, the QED-MP2 correction reads

$$E^{(2)}_{\text{HF},0} = -\sum_{\mu,n} \frac{\langle \mu, n|H_{\text{SC}}\,|\text{HF}, 0\rangle\,\langle \text{HF}, 0|H_{\text{SC}}\,|\mu, n\rangle}{E^{(0)}_{\mu,n} - E^{(0)}_{\text{HF},0}} \quad ; \quad \mu, n \neq \text{HF}, 0. \tag{SE58}$$

In this last equation we substituted V with H_{SC} because of eq. (SE53) and the orthogonality of the basis states in eq. (SE46). Because of the hermicity of the Hamiltonian, we can rewrite this correction in the following manner

$$E^{(2)}_{\text{HF},0} = -\sum_{\mu,n} \frac{|\langle \text{HF}, 0|H_{\text{SC}}\,|\mu, n\rangle\,|^2}{E^{(0)}_{\mu,n} - E^{(0)}_{\text{HF},0}} \quad ; \quad \mu, n \neq \text{HF}, 0. \tag{SE59}$$

Now we go through each type of excitation that contributes to the correction in eq. (SE59) and, for each of them, we see which terms of the SC-Hamiltonian in eq. (SE41) do contribute. The photon energy $\omega b^\dagger b$ obviously never contributes, while for evaluation of the denominators we use eq. (SE54).

1. We first start by considering purely photonic excitations ($\mu = 0,\ n > 0$). The $n = 1$ contribution is zero because of the Brillouin condition in the photonic part

$$\langle \mathrm{HF}, 0 | \left[H_{\mathrm{SC}}, \tilde{E}_{pp}(b - b^\dagger) \right] | \mathrm{HF}, 0 \rangle = 0. \tag{SE60}$$

The SC-transformed electronic-like Hamiltonian

$$H_{el}^{\mathrm{SC}} = \sum_{pq} \tilde{h}_{pq}^{\mathrm{SC}} Y_{pq} \tilde{E}_{pq} + \frac{1}{2} \sum_{pqrs} \tilde{g}_{pqrs}^{\mathrm{SC}} Y_{pqrs} \tilde{e}_{pqrs} \tag{SE61}$$

contributes for all the $n > 1$. Specifically

$$\sum_{n=2}^{\infty} \frac{|\langle \mathrm{HF}, 0 | H_{el}^{\mathrm{SC}} | \mathrm{HF}, n \rangle|^2}{E_{\mathrm{HF},n}^{(0)} - E_{\mathrm{HF},0}^{(0)}} = \sum_{n=2}^{\infty} \frac{(E^n)^2}{n\omega}, \tag{SE62}$$

where

$$E^n = 2 \sum_i h_{ii}^n + \sum_{ij} (2 g_{iijj}^n - g_{ijji}^n) \tag{SE63}$$

and the the nth one and two electron integrals in the canonical basis depend from the Laguerre polynomials obtained by calculation of the displacement Franck-Condon factors

$$h_{pq}^n = \frac{1}{\sqrt{n!}} \sum_{rs} C_{pr}\ \tilde{h}_{rs}^{\mathrm{SC}}\ Q_{rs} \left(\frac{\lambda}{\sqrt{2\omega}} (\eta_r - \eta_s) \right)^n C_{qs} \tag{SE64}$$

$$g_{pqrs}^n = \frac{1}{\sqrt{n!}} \sum_{tuvz} C_{pt} C_{rv}\ \tilde{g}_{tuvz}^{\mathrm{SC}}\ Q_{tuvz} \left(\frac{\lambda}{\sqrt{2\omega}} (\eta_t - \eta_u + \eta_v - \eta_z) \right)^n C_{qu} C_{sz}. \tag{SE65}$$

2. Secondly, we consider single (S) excitations in the orbital space coupled with generic photonic excitations ($\mu \in S,\ n > 0$). The $n = 0$ contributions are zero because of the

Brillouin condition in the orbital part

$$\langle \mathrm{HF}, 0| \left[H_{\mathrm{SC}}, E_{ai}^{-}\right] |\mathrm{HF}, 0\rangle = 0. \tag{SE66}$$

Using Slater-Condon rules for single excited determinants, we obtain

$$\sum_{\mu \in S} \sum_{n=1}^{\infty} \frac{|\langle \mathrm{HF}, 0| H_{\mathrm{SC}} |\mu, n\rangle|^2}{E_{\mu,n}^{(0)} - E_{\mathrm{HF},0}^{(0)}} = \sum_{ai} \sum_{n=1}^{\infty} \frac{(F_{ai}^{n})^2}{n\omega + \epsilon_a - \epsilon_i}, \tag{SE67}$$

where the nth-Fock matrix elements read

$$F_{pq}^{n} = h_{pq}^{n} + \sum_{i} (2g_{pqii}^{n} - g_{piiq}^{n}) + \delta_{n1} \lambda \sqrt{\frac{\omega}{2}} ((\tilde{\mathbf{d}} \cdot \boldsymbol{\epsilon})_{pp} - \eta_p). \tag{SE68}$$

Again we made use of the displacement Franck-Condon factors:

$$\langle n| e^{-\alpha(b - b^{\dagger})} |m\rangle = \begin{cases} \sqrt{\frac{m!}{n!}}\, \alpha^{n-m} e^{-\alpha^2/2} L_m^{n-m}(\alpha^2), & n \geq m \\ \sqrt{\frac{n!}{m!}}\, (-\alpha)^{m-n} e^{-\alpha^2/2} L_n^{m-n}(\alpha^2), & n < m \end{cases} \tag{SE69}$$

where L_p^q is the Laguerre q-th order polynomial of degree p.

3. Lastly, we consider doubly (D) excitations in the orbital space coupled with generic photonic excitations ($\mu \in D,\ n > 0$). In this case, all the $n > 0$ do contribute. No more than double excitation need to be considered because, according to Slater-Condon rules, the SC-Hamiltonian in eq. (SE41) cannot connect states differing by more than two occupied orbitals. Only the two electron part of the SC-transformed electron-like Hamiltonian in eq. (SE61) do contribute. Analogously to standard MP2 theory, this correction reads

$$\sum_{\mu \in D} \sum_{n=0}^{\infty} \frac{|\langle \mathrm{HF}, 0| H_{\mathrm{SC}} |\mu, n\rangle|^2}{E_{\mu,n}^{(0)} - E_{\mathrm{HF},0}^{(0)}} = \sum_{aibj} \sum_{n=0}^{\infty} \frac{g_{aibj}^{n}(2g_{aibj}^{n} - g_{ajbi}^{n})}{n\omega + \epsilon_a - \epsilon_i + \epsilon_b - \epsilon_j}. \tag{SE70}$$

So, the second order energy correction to the SC ground-state is

$$E^{(2)} = -\sum_{n=2}^{\infty} \frac{(E^n)^2}{n\omega} - \sum_{n=1}^{\infty}\sum_{ai} \frac{(F^n_{ai})^2}{n\omega + \epsilon_a - \epsilon_i} - \sum_{n=0}^{\infty}\sum_{aibj} \frac{g^n_{aibj}(2g^n_{aibj} - g^n_{ajbi})}{n\omega + \epsilon_a + \epsilon_b - \epsilon_i - \epsilon_j}. \quad \text{(SE71)}$$

By capturing electron-photon correlation, this MP2 correction is correctly non size-extensive when considering two subsystems largely separated within a cavity.

References

(1) Berestetskii, V. B.; Lifshitz, E. M.; Pitaevskii, L. P. *Quantum Electrodynamics: Volume 4*; Butterworth-Heinemann, 1982; Vol. 4.

(2) Helgaker, T.; Jorgensen, P.; Olsen, J. *Molecular electronic-structure theory*; John Wiley & Sons, 2013.

(3) Cohen-Tannoudji, C.; Dupont-Roc, J.; Grynberg, G. *Photons and atoms: introduction to quantum electrodynamics*; John Wiley & Sons, 2024.

(4) Riso, R. R.; Haugland, T. S.; Ronca, E.; Koch, H. Molecular orbital theory in cavity QED environments. *Nat. Commun.* **2022**, *13*, 1368.

(5) El Moutaoukal, Y.; Riso, R. R.; Castagnola, M.; Koch, H. Toward polaritonic molecular orbitals for large molecular systems. *J. Chem. Theory Comput.* **2024**, *20*, 8911–8920.
